# Coarctation of the aorta in a young female treated with left subclavian artery to descending thoracic aorta bypass: a case report

**DOI:** 10.1590/1677-5449.202200182

**Published:** 2023-01-13

**Authors:** Kishore Abuji, Venkata Vineeth Vaddavalli, Deepak Kumar, Naveen Maheshwari, Ujjwal Gorsi, Lileswar Kaman, Ajay Savlania

**Affiliations:** 1 Post Graduate Institute of Medical Education and Research - PGIMER, Chandigarh, India.

**Keywords:** coarctation of aorta, left subclavian artery to descending thoracic aorta bypass, hypertension, congenital heart disease, coartação da aorta, artéria subclávia esquerda para derivação de aorta torácica descendente, hipertensão, cardiopatia congênita

## Abstract

Coarctation of the aorta is a rare congenital abnormality, with an incidence of 6-8% of all congenital heart problems. It is usually diagnosed in childhood during routine clinical examination and adults mostly present with hypertension. Various investigations like transthoracic echocardiography, computed tomography, and magnetic resonance angiography can help with diagnosis. Prognosis depends on age at presentation and the severity of coarctation. Treatment options available are open and endovascular repair. Extra-anatomical bypass is the preferred option in cases with unfavorable anatomy. Long term follow up is required post repair due to risk of restenosis and aneurysm formation. Here is a case in which a young female presented with hypertension, was diagnosed with coarctation of the aorta, and was treated a left subclavian artery to descending thoracic aorta bypass. Her postoperative course was uneventful and she had improvement in hypertension.

## Resumo

A coarctação da aorta é uma anomalia congênita rara, com incidência de 6-8% entre todos os problemas cardíacos congênitos. É geralmente diagnosticada na infância durante o exame clínico de rotina, e os adultos normalmente apresentam hipertensão. Diversas investigações, como ecocardiografia transtorácica, tomografia computadorizada e angiorressonância magnética, podem auxiliar no diagnóstico. O prognóstico depende da idade na apresentação e da gravidade da coarctação. As opções de tratamento disponíveis são o reparo aberto e o reparo endovascular. O *bypass* extra-anatômico é a opção preferida em caso de anatomia desfavorável. O acompanhamento de longo prazo é necessário após o reparo devido ao risco de reestenose e formação de aneurisma. Descrevemos o caso de uma jovem do sexo feminino que apresentou hipertensão, a qual foi diagnosticada como coarctação da aorta. Foi realizada derivação da artéria subclávia esquerda para a aorta torácica descendente. O pós-operatório transcorreu sem intercorrências, e a paciente apresentou melhora da hipertensão.

## INTRODUCTION

Coarctation of the aorta has an incidence of 6-8% of all congenital heart problems. A slight male predominance is seen.^1^ It is mostly diagnosed during childhood, often in the first few weeks of life in severe disease. Milder cases and those with patent ductus arteriosus can remain asymptomatic until adulthood, accounting for nearly 10% of adult patients with congenital heart disease.^2^ Upper extremity hypertension is the most common clinical presentation in adulthood, others being claudication, aortic aneurysm or dissection, and cerebrovascular disease.^3^ Here, we present a case of coarctation of the aorta in a young female who presented with hypertension and in whom a left subclavian artery to descending thoracic aorta bypass was done using 12mm tubular polyester graft.

The protocol was approved by the Institutional Ethics Committee (Post Graduate Institute of Medical Education and Research, INT/IEC/2021/001100).

## CASE PRESENTATION

**Figure 1:**
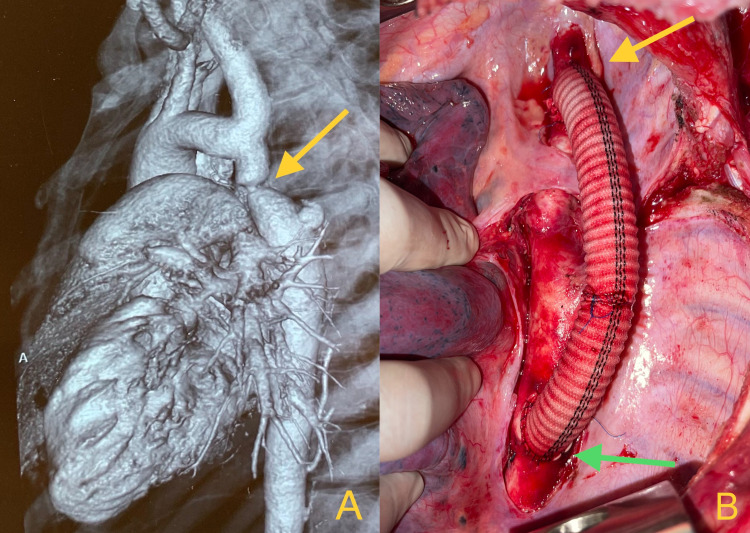
**(A)** CT volume rendered image (CRT) showing post subclavian coarctation of aorta with post stenotic dilatation of aorta (yellow arrow); **(B)** Intraoperative picture showing left subclavian artery (yellow arrow) to descending thoracic aorta bypass (green arrow) with polyester graft.

An 18-year female with no known comorbidities presented to the gynecology outpatient clinic with menorrhagia. On routine clinical examination, she was found to have severe hypertension of 170/100mmHg (bilateral upper limbs) and was started on antihypertensive medications. On examination, she had weaker pulses in the lower extremities (Femoral artery pulses) and her ankle systolic pressure was 80mmHg. Duplex examination of bilateral kidneys showed parvus tardus flow in renal arteries. Following this presentation, she underwent computed tomography (CT) angiography of the chest and abdomen, which showed severe coarctation of the aorta 2cm distal to the origin of the left subclavian artery, with post stenotic dilatation, and dilated intercostal arteries (Figure 1A). Chest X-ray showed classical rib notching. Echocardiography showed an ejection fraction of 55% with left ventricular hypertrophy without any valvular abnormalities. She was considered for a left subclavian artery to descending thoracic aorta bypass due to significant size disparity of distal arch and post stenotic dilated thinned out aorta (unfavorable anatomy). Routine blood workup was obtained and the patient was optimized for surgery.

**Figure 2:**
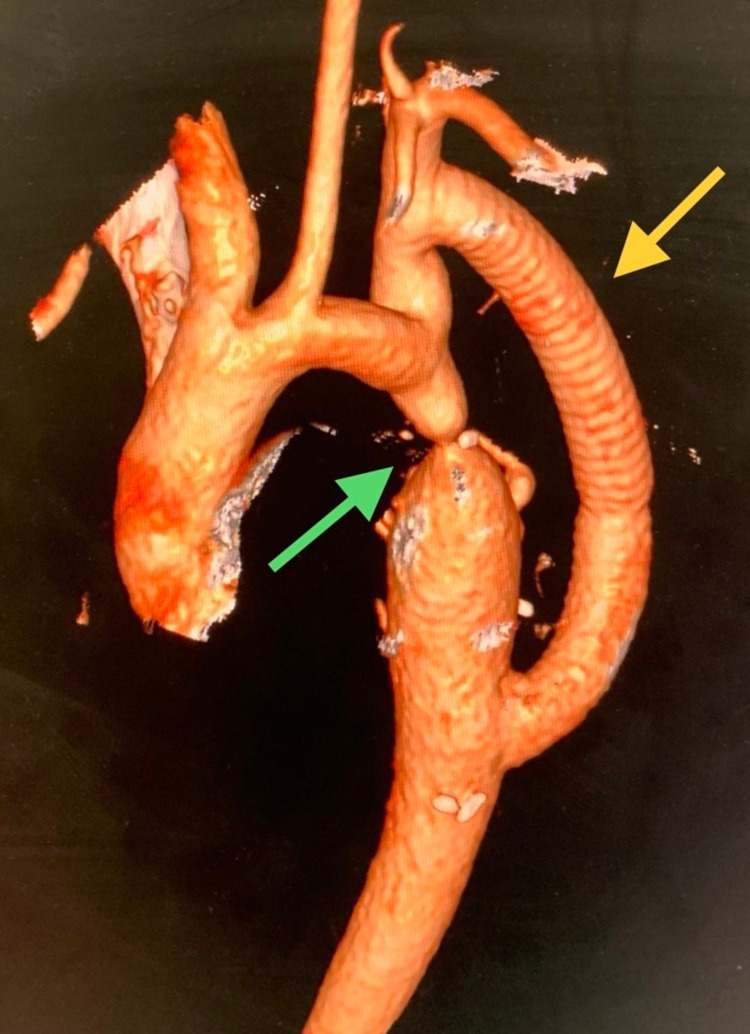
CT angiogram of the chest region, volume rendered image, showing left subclavian to descending thoracic aortic bypass (yellow arrow).The coarctation can also be seen (green arrow).

The patient was induced under general anesthesia using a double lumen tube (DLT) for single lung ventilation. In the right lateral position, a thoracotomy was performed through the left fourth intercostal space. Intraoperatively, multiple collaterals were present from intercostal arteries. Coarctation was present 1cm distal to the origin of the left subclavian artery. There was no ductus arteriosus. Left subclavian artery to descending thoracic aorta bypass was constructed using 12mm coated tubular polyester graft (Figure 1B, Figure 2). She had an unremarkable postoperative recovery and at discharge her blood pressure was equal in upper and lower limbs (130/80mmHg on Tab Metoprolol 12.5 mg). The imaging shown in Figure 2 was done at 1 month follow-up. She had normalized blood pressure in all four limbs at 3 months without any need for antihypertensive medication and is now doing well at 1 year follow-up. CT angiography was not performed at 1 year follow-up because the patient had good clinical evolution and was a young woman. At present, she has been kept on yearly follow up.

## DISCUSSION

Coarctation of the aorta (CoA) is defined as obstruction of the blood flow in the aorta. The incidence of CoA was 6-8% of all congenital heart diseases and it is more common in males.^1^ The presentation of CoA is variable based on age. Adolescents and adults mostly present with hypertension. The hypertension in patients with CoA is due to mechanical obstruction to ventricular ejection as well as hypotension induced activation of the renin angiotensin aldosterone system.^3^ In our case, the patient was diagnosed with CoA during workup for hypertension. Other clinical signs and symptoms may include bounding upper limb pulses, weak femoral pulses, lower limb claudication, and arm-leg blood pressure gradient of >20mmHg.^4^ Other manifestations are severe headaches, bicuspid aortic valve, neurological complaints, and cardiac failure. Nearly 10% of CoA are associated with berry aneurysms in the circle of Willis.^5^ Prognosis and survival depend upon age and severity of disease at the time of presentation. The cause of death in these patients is heart failure, aortic rupture/dissection, coronary artery disease, infective endocarditis, or cerebral hemorrhage.^6^^,^^7^ Transthoracic echocardiography is the first line of investigation to confirm the diagnosis of CoA. It identifies the anatomy of the arch and site of coarctation, determines the severity, and assesses intracardiac abnormalities.^8^ Computed tomography allows evaluation of structures in two dimensions and provides a reconstructed three-dimensional view. It is a great tool to assist with surgical planning. Other modalities to diagnose CoA are magnetic resonance (MR) angiography and invasive angiography. Multiple treatment options available to treat this condition are surgical repair, transcatheter balloon angioplasty, and transcatheter stent implantation. The preferred treatment option depends upon the anatomy of the coarctation, the patient’s age and comorbidities at the time of presentation, and the center’s experience. With the advancement of endovascular treatment and technology, adult patients are preferably treated with endovascular modalities.^9^ However, surgery remains a treatment option for all age groups. Surgical techniques include end-to-end anastomosis, extended end-to-end anastomosis, subclavian flap repair, interposition graft, and left subclavian artery to descending thoracic aorta bypass.^10^ In our case, we preferred left subclavian artery to descending thoracic aorta bypass over extended end-to-end anastomosis and endovascular stent graft implantation due to significant disparity in the size of distal arch and post stenotic dilated aorta. Elkerdany et al. described a total of twenty-two patients treated with left subclavian artery to DTA bypass using woven double velour graft. Their study showed a significant drop in the pressure gradient across the coarcted segment (p = 0.001) and a significant drop in systolic blood pressure (p = 0.009). There was no hospital mortality or major postoperative complications.^11^ Major postoperative complications include recoarctation, aortic aneurysm or dissection, and late systemic hypertension. The risk of recoarctation is comparable with both endovascular and surgical modalities in adults (6-9%). The risk of aortic pseudoaneurysm formation post open repair is 9%, most common with subclavian flap repair. The incidence of aneurysm formation with stent graft placement is lower (5%) than after balloon angioplasty (20%). Late systemic hypertension is found in 20-40% of adults post repair. Age at the time of repair is an important predictor of development of late systemic hypertension.^12^ Adults with CoA should be followed-up 6 to 12-monthly in the first year and then yearly with BP in all four limbs and cross-sectional imaging of repair site. MRA is preferred over CTA for follow-up in view of cumulative radiation exposure and can be undertaken 5-yearly if the first post repair scan is reassuring. Follow-up should also include consultation with a cardiologist and evaluation for intracranial aneurysms.^13^ One limitation of this case report is the lack of a 1 year follow-up control image.

## CONCLUSION

Coarctation of the aorta is a rare congenital cardiac abnormality. If untreated, patients rarely survive to old age. The treatment options will depend upon age of presentation, site of coarctation, and comorbidities. Extraanatomical left subclavian artery to descending thoracic aorta bypass is a good option to treat coarctation of the aorta in cases where anatomy is not favorable for endovascular stenting or direct distal arch repair.
